# William Beaumont—Memorial Address

**Published:** 1902-09

**Authors:** Robert Luedeking

**Affiliations:** St. Louis


### CORRESPONDENCE.

### William Beaumont—Memorial Address.

*Editor Buffalo Medical Journal:*

Sir.—The undersigned committee takes pleasure in announcing, that Prof. William Osler, of Johns Hopkins University, Baltimore, will deliver a memorial address on "William Beaumont, the First and Greatest American Physiologist," under the auspices of the St. Louis Medical Society of Missouri. The lecture takes place at the Odeon, on Saturday, October 4, 1902, at 8 o'clock p.m. The medical profession of your city and environment, is cordially invited to attend and to honor our distinguished guest.

The subject to be presented and the eminence of the essayist, make this occasion one of the greatest importance and interest. We ask your hearty coöperation, and beg of you to give this announcement the publicity that it merits.

For the committee,

ROBERT LUEDEKING, Chairman.

St. Louis, August 25, 1902.

